# 184. Inducible Clindamycin Resistance Testing on Pediatric *Streptococcus pneumoniae* Isolates

**DOI:** 10.1093/ofid/ofab466.184

**Published:** 2021-12-04

**Authors:** Liset Olarte, Douglas S Swanson, Jennifer E Tabakh, Dithi Banerjee, Rangaraj Selvarangan

**Affiliations:** 1 Children’s Mercy Kansas City, Kansas City, Missouri; 2 Children’s mercy Hospital, Kansas City, MO; 3 Children’s Mercy Hospital, Kansas City, Missouri

## Abstract

**Background:**

In 2013, the Clinical and Laboratory Standards Institute recommended inducible clindamycin resistance (ICR) testing on macrolide-resistant *Streptococcus pneumoniae* isolates, which arises due to the *erm*B gene. Ribosomal methylation by *erm*B confers resistance to macrolides (high-level resistance), lincosamides and streptogramin B. The goal of our study is to characterize the prevalence of ICR among pediatric pneumococcal isolates.

**Methods:**

We identified erythromycin-resistant(R) (minimum inhibitory concentration [MIC] ≥ 1 µg/mL) and clindamycin-susceptible(S) (MIC ≤ 0.25 µg/mL) pneumococcal isolates from pediatric patients seen at Children’s Mercy Hospital from 2007 to 2017. Determination of ICR was achieved via disk approximation (D-zone test) with standard erythromycin (15 µg) and clindamycin (2 µg) disks. Isolates with high-level erythromycin resistance (MIC ≥ 32µg/mL) were also tested for *erm*B gene by PCR. Positive and negative controls were used for D-zone test and *erm*B PCR.

**Results:**

We identified 289 erythromycin-R pneumococcal isolates; of those 194 (67.1%) were clindamycin-S (**Figure 1**). One-hundred and sixty-nine isolates were available for ICR testing, 166 (98%) isolates represented non-invasive disease samples. Median age of patients with erythromycin-R and clindamycin-S isolates was 19 (range 0.1 – 180) months. None of the isolates expressed ICR based on the D-zone test. Thirteen of those isolates (7.7%) expressed high-level erythromycin-R (MIC range 32-128 µg/mL); all were negative for *erm*B. The most common serotypes/serogroups among erythromycin-R and clindamycin-S isolates were: 15 (n=22), 35B (n=19), 11 (n=16), 6 (n=16), 19A (n=14) and 33 (n=12).

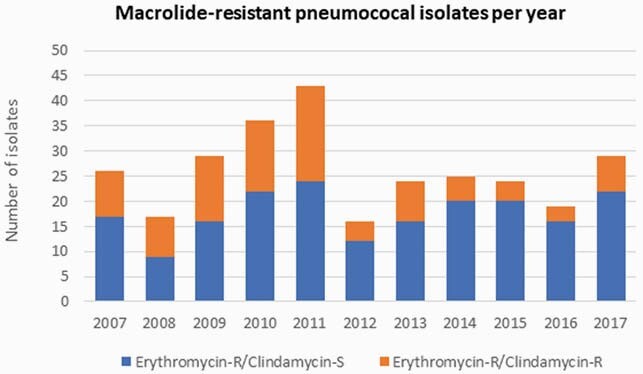

**Conclusion:**

Erythromycin-R and clindamycin-S pneumococcal isolates did not express ICR and isolates with high-level erythromycin-R did not carry *erm*B. Multicenter studies are needed to determine if ICR testing is required for macrolide-resistant pneumococcal isolates in the PCV13 era.

**Disclosures:**

**Liset Olarte, MD, MSc**, **GSK** (Research Grant or Support)**Merck** (Research Grant or Support)**Pfizer** (Research Grant or Support)**Sanofi** (Research Grant or Support) **Douglas S. Swanson, MD**, **Merck** (Research Grant or Support)**Pfizer** (Research Grant or Support)**Sanofi** (Research Grant or Support)

